# The Anatomical Remembrancer, or Complete Pocket Anatomist, &c. &c.

**Published:** 1846-03

**Authors:** 


*The Anatomical Remembrancer, or Complete Pocket Anatomist, &c. &c.,* containing a concise description of the Bones, Ligaments, Muscles, Viscera, &c. &c. From the Second London Edition, revised. New York: SAM'L S. & WM. WOOD, 1845, pp. 245.

This little work is designed to refresh the memory of the student, after his dissections and the study of more detailed works on the subject.

